# Mixed Reality–Based Physical Therapy in Older Adults With Sarcopenia: Preliminary Randomized Controlled Trial

**DOI:** 10.2196/76357

**Published:** 2025-08-01

**Authors:** Yeongsang An, Seunghwa Min, Chanhee Park

**Affiliations:** 1 Funrehab Co., Daejeon, Republic of Korea; 2 Department of Physical Therapy, Jeonju University, 303, Cheonjam-ro, Wansan-gu, Jeonbuk-do, Jeonju, 55069, Republic of Korea, 82 063-220-2375

**Keywords:** sarcopenia, mixed reality rehabilitation, older adults, artificial intelligence, digital health

## Abstract

**Background:**

Sarcopenia in older adults is associated with reduced muscle mass and function, leading to frailty, increased fall risk, and decreased quality of life (QOL). Mixed reality (MR)–based interventions have emerged as promising tools to enhance physical therapy engagement and effectiveness through immersive, interactive environments.

**Objective:**

This study aimed to investigate the effects of a Mixed Reality–Based Physical Therapy (Mr.PT) platform on quadricep muscle thickness, balance confidence, activities of daily living, and QOL in older adults with sarcopenia.

**Methods:**

A preliminary randomized controlled trial was conducted involving 30 older women (mean age 75.3, SD 9.9 y) diagnosed with sarcopenia based on the Asian Working Group for Sarcopenia criteria. Participants were randomly assigned to either the Mr.PT group or a conventional physical activity (CPA) group. Both groups participated in 30-minute exercise sessions, 3 times per week, over 4 weeks. The Mr.PT program used head-mounted MR devices with gamified, interactive training, while the CPA group received standard therapist-led exercises using resistance bands. Outcome measures included ultrasound imaging of quadricep muscle thickness, the Korean version of the Activities of Daily Living scale (Katz Index of Independence in Activities of Daily Living), the Activities-Specific Balance Confidence (ABC) scale, and the 12-Item Short-Form Health Survey. Statistical analysis was performed using repeated-measures ANOVA and Tukey post hoc tests.

**Results:**

The Mr.PT group showed significantly greater improvement in quadricep muscle thickness than the CPA group (*P*=.001). Within-group improvements in balance confidence (ABC scale) and daily functioning (Katz Index of Independence in Activities of Daily Living) were observed in both groups (*P*<.05), though between-group differences were not statistically significant. However, the Mr.PT group demonstrated significantly greater gains in QOL as measured by 12-Item Short-Form Health Survey (*P*=.02). All participants completed the intervention without dropouts or adverse events.

**Conclusions:**

MR-based exercise using the Mr.PT platform appears effective in increasing muscle mass and enhancing QOL among older adults with sarcopenia. Its interactive and adaptive features may improve engagement and motivation, suggesting potential advantages over traditional programs. Further research with larger cohorts and longer follow-up is recommended to confirm these preliminary findings and explore long-term outcomes.

## Introduction

### Background

Mixed reality (MR)–based digital therapeutics have emerged as a novel and promising technology for addressing the growing health care demands of aging populations [1]. Sarcopenia, defined as the progressive loss of skeletal muscle mass and strength with advancing age, represents a significant geriatric concern associated with frailty, increased fall risk, impaired activities of daily living (ADL), and diminished quality of life (QOL) [2,3]. Quadricep muscle thickness is a reliable indicator of lower-limb strength, which is essential for mobility and fall prevention. Impairments in balance confidence and ADL are commonly associated with sarcopenia and contribute to loss of independence. Additionally, QOL often deteriorates in this population due to physical limitations [2,3]. This geriatric issue is widespread and progressive and can lead to hospitalization [4-6].

### Rationale

Traditional interventions for sarcopenia, such as group-based or resistance exercise programs, offer clinical benefits but are limited by their standardized design, often requiring fixed schedules, group coordination, and in-person delivery [3,7]. These limitations were exacerbated during the COVID-19 pandemic, highlighting the urgent need for flexible, individualized therapeutic strategies [8,9]. Moreover, conventional group exercise typically lacks sufficient personalization, making it difficult to optimally engage and progress participants based on their functional capabilities.

To address these gaps, we developed a Mixed Reality–Based Physical Therapy (Mr.PT) platform designed to deliver personalized, engaging, and accessible exercise interventions. Mr.PT uses commercially available MR devices that integrate digital and real-world elements, enabling users to interact with digital environments while maintaining physical context [9,10]. Mr.PT comprises inexpensive, motivational, fun, and intensive devices [11]. The Mr.PT platform is designed to work with MetaQuest, and the software allows clinicians and clients free access to the platform without spatiotemporal constraints. The movement kinematics when reaching, grasping, transporting, and releasing objects in a digital environment are comparable to those in the physical world [12,13]. Unlike virtual reality (VR), which immerses the user in a fully simulated environment, MR overlays digital elements onto the real world and enables users to interact with both. This creates a more ecologically valid and functionally relevant experience, facilitating engagement and motor learning in rehabilitation settings [14]. Proprioceptive feedback has been suggested to exploit the multimodal aspects of correct active movements. However, clinical research on these systems has mainly focused on neurorehabilitation training rather than geriatric physical therapy.

### Study Aim

While MR-based interventions have been explored extensively in the fields of neurorehabilitation and motor recovery, evidence supporting their use in geriatric physical therapy—particularly for sarcopenia management—is scarce. Thus, this study aimed to evaluate and compare the effects of Mr.PT and conventional physical activity (CPA) programs on quadricep muscle thickness, balance confidence, ADL independence, and QOL among older adults with sarcopenia.

Given the accelerating trends of global aging, especially in countries like South Korea and Japan, there is an urgent need to develop accessible, scalable, and individualized rehabilitation interventions. MR-based platforms such as Mr.PT may offer sustainable solutions to enhance functional outcomes and support independent living among older adults.

## Methods

### Participants

A convenience sample of 30 older adults with sarcopenia (mean age 75.3, SD 9.9 y; all female) was recruited from a geriatric welfare center. Inclusion criteria were as follows: (1) 65 years and older, and (2) diagnosis of sarcopenia by a medical doctor based on established clinical criteria. Sarcopenia was diagnosed according to the 2019 criteria of the Asian Working Group for Sarcopenia, including low skeletal muscle mass (Skeletal Muscle Index<5.7 kg/m² by BIA), low muscle strength (grip strength <18 kg), and poor physical performance [15]. Basic demographic information (age, sex, height, weight, and BMI) was collected during baseline assessment using a standardized intake form completed by participants.

### Ethical Considerations

This study was approved by the Institutional Review Board of Jeonju University (Institutional Review Board number jjIRB-250206-HR-2024‐1203) prior to participant enrollment. All participants provided written informed consent before participation. All personal data were deidentified prior to analysis to ensure confidentiality and privacy. Only study ID numbers were used in the dataset. Participants received a small nonmonetary gift (health supplement worth approximately 30,000 KRW [US $23]) as compensation for their time. No personally identifiable images or data were included in the manuscript or supplementary materials. All ultrasound and evaluation images were anonymized. Although the clinical trial registration (KCT0010241) was finalized after study completion due to administrative delays, the study was retrospectively registered to ensure transparency. Recruitment and data collection were completed before the registration was officially approved.

### Randomization and Blinding

Participants were randomized into either the Mr.PT group or the CPA group using a simple coin-flip method. Randomization occurred prior to baseline assessments to minimize bias. Assessments were conducted by blinded evaluators who were unaware of group assignments, ensuring a single-blinded study design.

### Experimental Setup

A procedural checklist was used to standardize all experimental procedures. Equipment was calibrated daily before data collection. Baseline assessments included quadricep muscle thickness, the Activities-Specific Balance Confidence (ABC) scale, the Katz Index of Independence in Activities of Daily Living (KIADL), and the 12-Item Short-Form Health Survey (SF-12). Both groups were supervised by licensed physical therapists during exercise sessions. For the Mr.PT group, a computer technician was present during interventions to address potential technical issues but did not interact with participants or influence the sessions.

### Ultrasound Imaging Measurement of Quadricep Muscle Thickness

**Figure 1.**
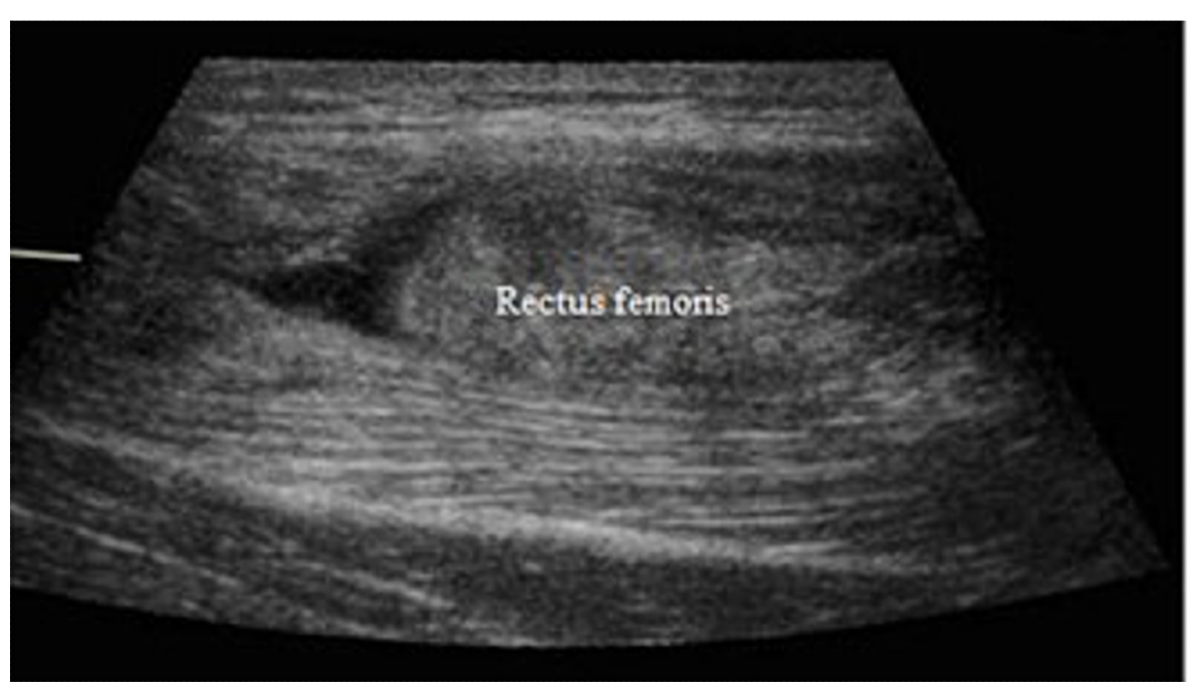
Rectus femoris ultrasound imaging.

An ultrasound probe (Bionet, Seoul, Republic of Korea) was used to obtain images of the rectus femoris muscle. All 2D ultrasound imaging acquisitions were acquired using an ultrasonographic system equipped with a single-sweep volumetric transducer. The apparatus had a linear vascular probe with a bandwidth of 3.3‐8.0 MHz, an aperture of 2.9 cm, and a maximum scanning depth of 8 cm, in addition to a phased array cardiac probe with a bandwidth of 1.7‐3.8 MHz and a field of view of 70° [16]. All participants were scanned while lying comfortably in the supine position. According to a previous study, the examiner positioned the probe on the anterior aspect of the quadriceps, perpendicular to its long axis, halfway between the proximal end of the patella and the anterior superior iliac spine. The examiner identified the rectus femoris, vastus intermedius, femur, and subcutaneous adipose tissues. To reduce artifacts, the skin was treated with excess gel. To examine the inter- and intra-rater reliability of the bilateral limbs, 3 examiners acquired pictures [16]. Among the 3 examiners were 3 physical therapists. The examiners were experts who had used ultrasound for evaluation in a clinical context for a minimum of 3 years. To prevent variations in the measurement and analysis of muscle data, each trial was performed by the 3 examiners separately within 2 hours of the initial assessment [16]. The top border of the rectus femoris was marked with a pointer on the screen of the ultrasound device (Figure 1).

This enabled the device to determine the total rectus femoris muscle thickness. Three measurements were made by each examiner to determine intrarater reliability [16,17]. The participants returned to their starting position after every investigation, and any gel or markings were removed from their skin by cleaning it. This minimized the possibility of measurement bias, such as anchoring, and guaranteed independent acquisition of each image and dataset.

### Activities-Specific Balance Confidence (ABC) Scale

An indirect indicator of falling risk, the ABC scale, was used to gauge the participants’ confidence in their ability to perform a range of ambulatory tasks without feeling unsteady or falling. The 16 ABC scale components have scores ranging from 0 (no confidence) to 100 (full confidence) [18]. The validity and reliability of the measurement tests have been thoroughly proven elsewhere [18].

### Katz Index of Independence in Activities of Daily Living

The best tool for determining a client’s functional status is a measure of their capacity to perform everyday tasks independently [19]. Age-related changes and health problems frequently result in a decline in the functional status of older adults. This decline often places older adults in a downward health spiral. An effective way to evaluate the health status of older adults is through their functional abilities [19]. An objective assessment that provides objective data helps indicate a decline or improvement in health status, allowing physical therapists to plan and intervene appropriately. The validity and reliability of the measurement tests have been thoroughly proven [19,20].

### Geriatric Quality of Life

Twelve items comprise the SF-12, which is a health-related QOL survey. Using this information, we evaluated 8 health areas, including mental and physical health [21]. General health, bodily discomfort, physical roles, and physical functioning were domains associated with physical health. Vitality, social functioning, emotional roles, and mental health are among the scales pertaining to mental health. The validity and reliability of outcome measurement tests have already been demonstrated in other contexts [22].

### Intervention

#### Overview

Both Mr.PT and CPA were assigned to participants in a randomized controlled trial, with a frequency of 30 min, and administered 5 times per week for 4 weeks. All participants were assessed using 2 (pre and post) evaluations.

#### Mr.PT Group

Participants in the Mr.PT group engaged in an MR-based exercise program using the MetaQuest head-mounted display system (Funrehab, Daejeon, Republic of Korea). The intervention was designed to simulate a variety of functional and cognitive tasks within an interactive digital environment that seamlessly integrated with real-world movement [12,23]. Each session began with participants standing or seated in a safe area, where they wore the MR device and received brief instructions on the day’s activities. Initial exercises involved evading digital objects approaching from various directions, requiring participants to shift their weight, adjust their posture, and take small steps to avoid collisions. This activity was intended to enhance dynamic balance, postural control, and lower limb reaction times [10]. The Mr.PT program included interactive MR tasks in which participants reached for and tracked digital objects projected through a head-mounted display. The speed of the digital objects ranged from 1.2 to 2.5 m/s, with target distances between 0.5 and 2.0 meters. The system automatically adjusted difficulty based on task success rates and provided real-time verbal and visual feedback.

**Figure 2.**
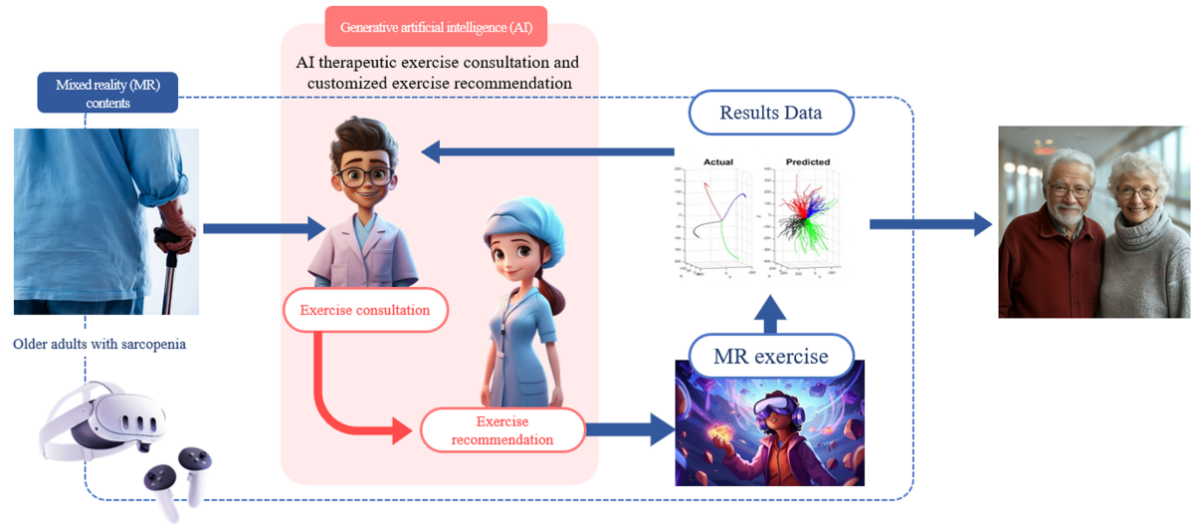
Mixed reality platform–based physical exercise therapy.

Subsequent tasks incorporated upper extremity movements, where participants were asked to “shoot” at digital targets appearing at random locations within their field of vision by performing rapid and precise shoulder abduction or flexion movements. This shooting task aimed to improve upper body coordination, reaction speed, spatial awareness, and proprioception [24]. As participants progressed through the program, combined tasks were introduced, simultaneously requiring both footwork (for dodging) and shoulder movements (for targeting) to complete challenges [25]. These integrated activities were specifically designed to train motor planning, divided attention, and cognitive-motor multitasking, which are often impaired in older adults with sarcopenia. The difficulty level of the digital tasks was automatically adjusted based on participant performance, ensuring an appropriate balance between challenge and skill to maintain motivation and maximize engagement (Figure 2) [26].

**Figure 3.**
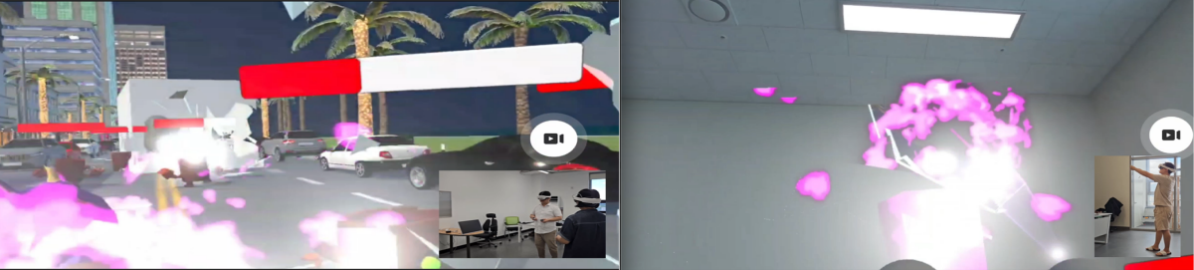
Mixed reality platform.

Verbal encouragement and visual feedback were provided throughout the sessions by the MR system, further supporting adherence and participant satisfaction. Each MR-based exercise session lasted 30 minutes and was conducted 5 times per week over the 4-week intervention period, under the supervision of a trained physical therapist and a computer technician who managed technical setup but did not influence participant behavior (Figure 3).

#### CPA Group

Participants assigned to the CPA group performed a conventional, therapist-led exercise program focused on maintaining and improving musculoskeletal strength, balance, and mobility. Sessions took place in a standard exercise room equipped with balance mats, chairs, and minimal resistance equipment [27]. Each CPA session consisted of warm-up activities such as marching in place and dynamic stretching, followed by structured moderate-intensity gait training across flat surfaces. Gait exercises incorporated variations in speed and direction to promote adaptability and coordination. Strength training exercises emphasized the major muscle groups responsible for functional mobility. Participants performed sets of hip flexion, extension, and abduction exercises, as well as knee flexion and extension drills, often using bodyweight resistance or elastic bands to adjust intensity [24]. Balance training involved static and dynamic activities, including one-legged standing with eyes open, tandem standing, and tandem walking along a straight line. Each exercise was modified according to individual ability, with progression achieved by increasing task complexity or duration. Upper extremity exercises targeted shoulder abduction, extension, and flexion using light dumbbells or resistance bands, aiming to maintain upper limb functional strength necessary for daily tasks. The CPA program consisted of therapist-supervised exercises using elastic resistance bands of light to medium strength (1.5‐3.0 kgf). Exercises included lower-limb strength, balance, and gait training with 10‐15 repetitions per exercise [14,28]. The Mr.PT group performed individualized MR-based tasks with real-time visual and verbal feedback, adjusting difficulty based on task success. The CPA group received therapist-led physical exercises using elastic resistance bands and balance training. All sessions were conducted individually to control dosage and engagement. Both interventions were conducted 3 times per week for 4 weeks. All 30 participants completed the intervention without dropouts. No adverse effects, such as dizziness or fatigue, were reported during or after the sessions.

### Statistical Analysis

Descriptive statistics (mean, SD) were calculated for all continuous variables. The Kolmogorov-Smirnov test confirmed normality assumptions. Repeated-measures ANOVA was used to compare pre- and postintervention changes within and between groups. Tukey’s post hoc tests were applied to examine significant interactions. The chi-square test was used for categorical data (eg, sex). Power analysis using G*Power 3.1.9.7 indicated that a sample size of 27 participants would achieve 80% power with an effect size of 0.5 (α=.05); thus, 30 participants were recruited to allow for potential dropout. Observed power was calculated post hoc for key outcomes. A significance threshold of *P*<.05 was used for all analyses. Statistical analyses were conducted using SPSS (version 26.0; IBM Corp) was used for the statistical analyses. A *P* value of .05 was determined.

## Results

**Figure 4.**
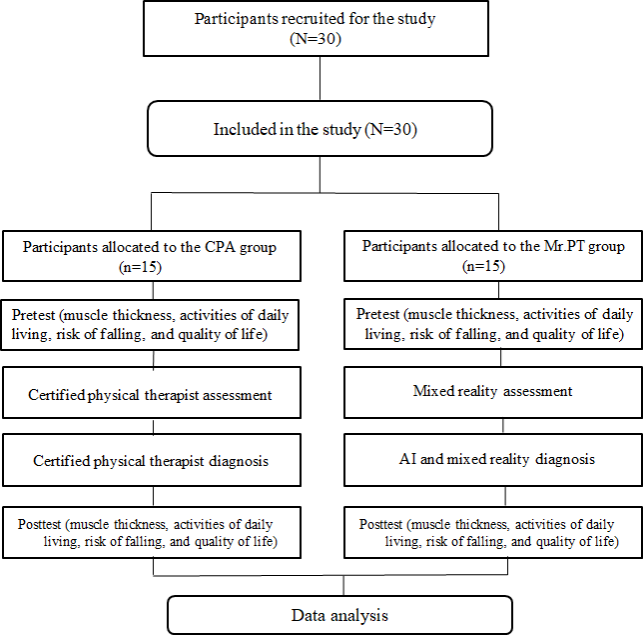
CONSORT (Consolidated Standards of Reporting Trials) flow chart. AI: artificial intelligence; CPA: conventional physical activity; Mr.PT: Mixed Reality–Based Physical Therapy.

Figure 4 shows the study flow chart.

### Demographic Data

Table 1 shows the demographic characteristics of the participants. Each participant completed all experimental tasks and exercises. The CPA and Mr.PT groups did not differ significantly in terms of sex, age, height, weight, or BMI (*P*>.05). 

**Table 1.** Participants’ demographic characteristics (N=30).

Characteristics	Mr.PT^a^ (n=15)	CPA^b^ (n=15)	*P* value
Age (years), mean (SD)	76.24 (8.7)	74.88 (9.1)	.68
Sex, n (%)			>.99
Male	0 (0)	0 (0)	
Female	15 (100)	15 (100)	
Height (cm), mean (SD)	153.12 (10.11)	155.88 (8.21)	.31
Weight (kg), mean (SD)	54.13 (6.13)	56.27 (4.88)	.45
BMI (kg/m^2^), mean (SD)	20.11 (1.81)	20.83 (0.91)	.23

a Mr.PT: Mixed Reality–Based Physical Therapy.

b CPA: conventional physical activity.

### Quadricep Muscle Thickness and KIADL Measurements

Repeated-measures ANOVA revealed a significant time effect (*P*=.001) for quadricep muscle thickness and KIADL, indicating improved muscle thickness and ADL for participants. No significant difference in KIADL scores was observed between the groups (*P*=.09). The interaction post hoc test revealed a greater improvement in quadricep muscle thickness in Mr.PT than in CPA, suggesting that quadricep muscle thickness is related to sarcopenia after Mr.PT than in CPA (Table 2).

**Table 2.** Quadricep muscle thickness and Katz Index of Independence in Activities of Daily Living (KIADL) measurement.

	Mr.PT^a^, mean (SD)		CPA^b^, mean (SD)		*P* value		
	Pretest	Posttest	Pretest	Posttest	Time effect	Between groups	Time × group
Quadricep muscle thickness (mm)	15.83 (1.83)	17.13 (1.77)	15.71 (2.01)	16.22 (1.38)	.001^c^	.02^c^	.001^c^
KIADL	3.90 (0.32)	4.20 (0.43)	3.80 (0.37)	4.10 (0.41)	.001^c^	.09	.08

a Mr.PT: Mixed Reality–Based Physical Therapy.

b CPA: conventional physical activity.

c *P*<.05.

### ABC Scale and SF-12 Measurements

Repeated-measures ANOVA revealed a significant time effect (*P*=.001) for the ABC scale and SF-12, indicating improved fall confidence and QOL for participants. No significant difference in the ABC scale scores was observed between the groups (*P*=.23). The interaction post hoc test revealed a greater improvement in SF-12 in Mr.PT compared with CPA, suggesting that QOL is related to sarcopenia after Mr.PT compared with CPA (Table 3).

**Table 3.** Activities-Specific Balance Confidence (ABC) scale and 12-Item Short-Form Health Survey (SF-12).

	Mr.PT^a^, mean (SD)		CPA^b^, mean (SD)		*P* value		
	Pretest	Posttest	Pretest	Posttest	Time effect	Between groups	Time × group
ABC scale	1223.48 (253.12)	1372.18 (178.55)	1218.68 (188.12)	1349.88 (163.88)	.001^c^	.23	.41
SF-12	60.84 (4.88)	74.20 (7.88)	63.18 (3.78)	74.10 (8.31)	.001^c^	.02^c^	.04^c^

a Mr.PT: Mixed Reality–Based Physical Therapy.

b CPA: conventional physical activity.

c *P*<.05.

## Discussion

### Principal Findings

The Mr.PT intervention led to a 9.1% increase in quadricep muscle thickness, which aligns with previous studies on immersive VR-based rehabilitation. While functional gains such as gait speed or grip strength were not assessed, literature suggests that each 1 mm increase in quadricep thickness may correspond to approximately a 5% increase in knee extensor force. The improvement in SF-12 (4.68%) exceeds the established minimal clinically important difference of 3 points, supporting its clinical relevance [29,30]. Although Mr.PT showed superior effects in muscle thickness and QOL, it is important to recognize the practical advantages of the CPA program. CPA involves simple, therapist-guided physical activity using minimal equipment, making it highly scalable and cost-effective for widespread community-based use. The lack of significant between-group differences in ADL and balance confidence (ABC) may reflect the short intervention duration or the limited sensitivity of the assessment tools to detect small changes. Alternatively, it is possible that CPA, despite its simplicity, was equally effective in maintaining these functions in the short term.

### Comparison to Prior Work

The quadricep muscle thickness–related sarcopenia analysis demonstrated that the improvement in muscle thickness (9.1%) was significantly different between the groups. This finding is consistent with those of the previous studies. As previously reported, increased muscle strength (10.2%) was observed using VR compared with the control group in 30 participants with sarcopenia [31]. As previously reported, increased muscle parameters (8.8%) were observed using an augmented reality-based exercise program compared with the control group of 30 participants with sarcopenia [32].

VR training has shown promising results in improving physical function and muscle strength in older adults. An 8-week unsupervised VR exercise program significantly enhanced hip muscle strength and balance control in older adults compared with a control group [33]. Similarly, a VR fitness program improved muscular fitness and body composition in young untrained men, although some measures showed less improvement than traditional exercise [34]. VR training also increased lower extremity muscle activation in older participants after an 8-week intervention using a Wii Fit [24]. Previous studies have highlighted the potential of MR technologies to enhance engagement, cognitive-motor integration, and exercise adherence in various populations. As previously demonstrated, muscle thickness assessed by ultrasound can effectively estimate knee extension muscle strength in females [35]. Our findings align with this growing body of evidence and suggest that MR-based exercise interventions may offer meaningful advantages for older adults at risk of sarcopenia.

The activities of daily living analysis demonstrated that the improvement in KIADL (7.7% and 7.9%, respectively) was significantly different pre- and postintervention in both groups. This finding is consistent with those of the previous studies. Liao et al [36] found increased ADL function (7.73%) using VR compared with the control group in 34 older adults with mild cognitive impairment. As previously reported, increased ADL (10.23%) was observed using VR-based applications compared with the control group in older people [37]. As previously found, improved executive function was observed using a VR-based program (22.5%) compared with the control group in 50 community-dwelling older individuals [27]. Gamification platforms increase user loyalty by providing highly immersive, engaging, and entertaining digital environments for various locations and challenges. Consistent with previous analyses, our findings imply that the application of commercial VR systems, including the Xbox Kinect, PlayStation, and Nintendo Wii, may be advantageous and practical for enhancing functional daily living in geriatrics [38,39].

We found that MR was more effective and provided more significant evidence in the field of occupation and ambulation in ADL performance [23,25,26]. VR has emerged as a promising tool for cognitive rehabilitation and training in ADL for patients with neurological disorders. Studies have shown that digital interventions can improve cognitive functioning, attention, memory, and executive function in patients with stroke [5,13]. VR systems allow for personalized progressive training in ADL tasks by providing real-time feedback to patients. These systems offer immersive and realistic environments that simulate everyday activities, making rehabilitation more engaging and motivating for users [40]. Research has also demonstrated the feasibility and usability of VR-based ADL training, with handheld controllers proving to be more intuitive and less demanding than traditional mouse inputs [41]. Overall, VR technology has great potential for enhancing cognitive rehabilitation by offering ecologically valid and personalized training experiences that can effectively supplement conventional therapeutic methods.

The fall confidence analysis demonstrated that the improvement in the ABC scale (12.15% and 10.97%) was significantly different pre and postintervention in both groups. This finding is consistent with those of the previous studies. Using Xbox gaming, as previously found, decreases the risk of falling (7.91%) compared with the control group in 60 older individuals [42]. People find VR games emotionally and physically taxing, and when playing, the players’ motions constantly change direction, pace, and speed. Therefore, rapid brain processing, adjustments, and control of body balance are required [26,41]. In addition, older adults can effectively use safe and reasonably priced technologies in their homes [43]. VR technology is promising for fall prevention and improvement in confidence in older adults. VR balance exercise programs can reduce the risk of falling and fear of falling in community-dwelling women [44]. A study using a balance rehabilitation unit found significant improvements in balance parameters, reduced falls, and lower fear of falling in older adults with a history of falls [45]. However, the effectiveness of VR interventions depends on user acceptance. Research on VR headsets for fall prevention has found that the intention to use was positively predicted by perceived usefulness, enjoyment, and ease of use [46].

QOL analysis demonstrated that the improvement in SF-12 (4.68%) was significantly different between the groups. This finding is consistent with those of the previous studies. As previously reported, an improved QOL (15.9%) was observed using VR compared with a control group in 60 older adults [43]. As previously found, an increased QOL (0.37%) was observed using a semi-immersive VR intervention compared with the control group in 100 older adults [47]. VR-based interventions have shown promising results in improving older adults’ QOL. Studies have demonstrated that VR can enhance the physical, cognitive, psychological, and social well-being of this population [47]. MR-based exercises have been found to significantly improve overall QOL, mental and physical health, social relationships, and depression symptoms in older adults [13,23]. While VR, augmented, and MR applications have become more developed in the physical and cognitive domains, research in the areas of psychological and social well-being is still emerging [48]. These findings suggest that digital therapeutic technology has a significant potential to enhance various aspects of older adults’ QOL. The superior outcomes in the Mr.PT group may be attributed to enhanced cognitive engagement, task variability, and the real-time feedback provided by the MR environment, which may elicit greater neuromuscular activation compared with the more repetitive nature of CPA.

### Limitations

This study has several limitations that should be addressed in future research. First, the sample consisted only of older women, which limits generalizability. Second, ultrasound-based measurements may involve some subjectivity despite being conducted by trained personnel. Third, the 4-week intervention duration may have been insufficient to produce long-term or functional improvements beyond muscle hypertrophy. Although we did not directly assess neurophysiological changes, it is plausible that MR-related improvements are partly mediated by cortical or subcortical adaptations. Confounders such as participant motivation, novelty effects, and attentional demand may also have influenced outcomes. Future studies should consider longer interventions and include more diverse participants and direct measures of functional strength.

### Conclusion

Clinical research has indicated that MR platform-facilitated therapeutic exercise is beneficial for improving muscle thickness, ADL, fall confidence, and QOL in older adults with sarcopenia. Our results provide therapeutic evidence that Mr.PT improves the recovery of quadricep muscle thickness and QOL in patients with depression and anxiety. Most importantly, the Mr.PT platform allows autonomous provision of real-time audiovisual feedback and effective training, which could serve as a basis for advanced rehabilitation sciences and medical evidence.

## Supplementary material

10.2196/76357 Checklist 1 CONSORT (Consolidated Standards of Reporting Trials) checklist.

## Abbreviations

ABC: Activities-Specific Balance Confidence
ADL: activities of daily living
CPA: conventional physical activity
KIADL: Katz Index of Independence in Activities of Daily Living
MR: mixed reality
Mr.PT: Mixed Reality–Based Physical Therapy
QOL: quality of life
SF-12: 12-Item Short-Form Health Survey
VR: virtual reality
